# Psychosocial problems in traumatized refugee families: overview of risks and some recommendations for support services

**DOI:** 10.1186/s13034-017-0210-3

**Published:** 2018-01-11

**Authors:** J. M. Fegert, C. Diehl, B. Leyendecker, K. Hahlweg, V. Prayon-Blum, the Scientific Advisory Council of the Federal Ministry of Family Affairs, Senior, Citizens, Women and Youth, Joerg M. Fegert, Margarete Schuler-Harms, Martin Werding, Sabine Andresen, Miriam Beblo, Claudia Diehl, Martin Diewald, Heiner Fangerau, Irene Gerlach, Kurt Hahlweg, Michaela Kreyenfeld, Birgit Leyendecker, Katja Nebe, Notburga Ott, Thomas Rauschenbach, C. Katharina Spieß, Sabine Walper

**Affiliations:** 1 Child and Adolescent Psychiatry/Psychotherapy, University Hospital Ulm, Steinhoevelstrasse 5, 89075 Ulm, Germany; 2 University of Konstanz, Konstanz, Germany; 3 Ruhr-Universitaet Bochum, Bochum, Germany; 4 Technische Universitaet Braunschweig, Braunschweig, Germany

**Keywords:** Post-traumatic stress, Psychosocial risk, Refugees, Families, Children, Support

## Abstract

This article is an abridged version of a report by an advisory council to the German government on the psychosocial problems facing refugee families from war zones who have settled in Germany. It omits the detailed information contained in the report about matters that are specific to the German health system and asylum laws, and includes just those insights and strategies that may be applicable to assisting refugees in other host countries as well. The focus is on understanding the developmental risks faced by refugee children when they or family members are suffering from trauma-related psychological disorders, and on identifying measures that can be taken to address these risks. The following recommendations are made: recognizing the high level of psychosocial problems present in these families, providing family–friendly living accommodations, teaching positive parenting skills, initiating culture-sensitive interventions, establishing training programs to support those who work with refugees, expanding the availability of trained interpreters, facilitating access to education and health care, and identifying intervention requirements through screening and other measures.

## Background

Refugees who have fled from war zones are at significantly increased risk for post-traumatic stress syndrome (PTSD) and other trauma-related disorders, which may lead to dysfunctional behaviors that impair their ability to cope with social and/or family life. Often, these behaviors burden the entire family system of those affected and complicate the already great challenges of integration into a new society. Hence, it is important that treatment be provided as early as possible.

Of the waves of refugees entering Europe in recent years, around 1.5 million applied for asylum in Germany between 2013 and 2016 [1], approximately one-third of them minors. In Germany, matters of family policy are handled by the Federal Ministry of Family Affairs, Senior Citizens, Women and Youth. The Ministry has established an independent Scientific Advisory Council made up of an interdisciplinary panel of experts. The Council’s latest report [2] addresses the high need for support for refugee families, with a focus on the psychosocial problems experienced by asylum seekers who have fled from war zones. This article presents an abridged version of that report, omitting details that are specific to the German health system and asylum laws, and including just those insights and recommendations that could be applied to other host countries as well.

At the peak of the so-called refugee crisis in Germany, there was an energetic and resourceful *Willkommenskultur* (welcoming culture) with regard to the newcomers and a widespread willingness to help them; however, recent surveys (see overview in [3]) indicate that public attitudes have hardened. This shift in attitude was possibly triggered, among other things, by a widely publicized incident that took place in Cologne on New Year’s Eve 2016, involving mass sexual assaults on women by organized groups of young men who appeared to mainly be from North Africa. In the public outcry that followed, the previously unquestioned fact that refugees suffer from extraordinarily high levels of psychological stress was challenged, along with resentment of the resources that were being directed toward assisting them. Several problematic attitudes came to be widely held: mental disorders, especially PTSD, were unjustly referred to as questionable illnesses that were being feigned in order to prevent deportation; and trauma-related symptoms of mental stress that are often present in refugees, such as panic attacks, sleep disorders, depression, and suicidality, were trivialized. Such downplaying raises the risk that government policies will follow an ill-advised direction when it comes to the provision of mental health resources. There is good empirical evidence for the effectiveness of psychosocial interventions in children and adolescents who have been exposed to traumatic experiences, whether these involved violence or natural disasters [4]; and curtailing these interventions could lead to serious short- and long-term disadvantages not only for those affected but also for the society that has taken them in.

Against this background, the Advisory Council set out to better understand the circumstances around traumatized refugee families and to determine what services are needed to assist them. It should be noted that the call here for easier access to mental health care services in order to reduce potential risks as early as possible is mirrored in a call for action by the European Society for Child and Adolescent Psychiatry in its position statement on the mental health of child and adolescent refugees [5].

The remainder of this article describes the psychosocial problems facing people fleeing from war zones and the dynamics within families in which one or more members have been affected by trauma; discusses what types of support services should be established in order to help refugee families adapt to their new environment and to treat post-traumatic disorders; and provides some concrete recommendations.

## Main text

### The psychosocial situation of refugee families from war zones

#### Mental disorders in refugees

It goes without saying that not all individuals who have lived through potentially traumatizing events will suffer afterwards from PTSD or other mental health problems; however, the risk for an increased incidence of such disorders, especially among children, is well documented in the literature (for a review, see [6]). Many refugee children have already been traumatized in their country of origin, whether by war-related events, social violence, or abuse within their own families, and many have been further exposed to life-threatening situations during their flight (for example, surviving a perilous crossing of the Mediterranean, or encountering dangerous situations in the country of destination). In general, acts of violence such as rape, torture, and armed conflict have far more devastating effects on their victims than do natural disasters or accidents. In both cases, however, the likelihood of developing PTSD increases with the number of traumatic events, with more exposure to trauma leading to a cumulative increase in both the likelihood and severity of this disorder. This finding applies to adults and children alike [7, 8].

Refugees from war zones have often faced a range of stress factors that are experienced by no other population. In their home country, traumatic experiences may have included bombs, imprisonment, torture, and exile; and for children, they often also include witnessing or being targets of domestic violence. Apart from the events that led to the flight abroad, the journey itself is often fraught with danger; and once in the country of exile, life is often characterized by insecure residency status, unemployment, poor housing conditions, and the challenges of learning a new language and integrating into a foreign culture [9].

Not surprisingly, such a high burden of stress can lead to psychological problems. At present, it cannot be stated with certainty what the prevalence of mental disorders is among refugees in Germany, but preliminary findings of a study in Syrian children in a refugee camp [10] found PTSD in 26% of those aged 6 years and younger and in 33% of 7- to 14-year-olds. Similarly, a study looking at a population of children aged 1 to 5 years whose families had fled from war zones in Iraq and Syria [11] found that one-third displayed symptoms indicating PTSD, with particularly high scores in the categories of anxiety/depression, social withdrawal, and attention deficits compared to the clinical reference samples. These figures correspond to what has been reported in international studies on the prevalence of mental disorders in refugees: Fazel et al. [12] found a prevalence rate of mental disorders ten times higher in samples of refugees settled in western countries, including depression disorders other than PTSD. In comparison, the rate of trauma-related disorders such as PTSD is just 3% in the general German population [13]. Overall, it is assumed that approximately 50% of refugees suffer from some form of mental disorder [14–16]. It must be noted that the diagnostic criteria for PTSD are less likely to be fulfilled by children than by adults: in children, the reaction to trauma is often to exhibit developmental regressions or delays, behavioral disorders, or other symptoms of stress [17].

If PTSD is left untreated, in about one-third of cases the condition becomes chronic [18, 19]. In particular, survivors of war and other forms of organized violence, both soldiers and civilians, are known to still suffer from psychological impairments years after the traumatic events [20]. In the case of children, who are among the most vulnerable, family circumstances may play an important role. In Germany, children and adolescents fleeing war zones who arrived without family members, referred to as “unaccompanied minor refugees”, became the focus of a great deal of professional and public attention, and studies on their specific psychological needs have been conducted [6, 21]. Until they reach the age of majority, these minors are granted almost the same rights that are available to their German peers, and are cared for by institutions for youth welfare, including full access to medical and psychotherapeutic services (apart from the limitations due to wait lists). However, for the more than 80% of child refugees who arrived in the company of their parents [22], the situation may be different.

#### Children with traumatized family members

Refugee children who have experienced traumatizing events in their home country, and possibly during their flight as well, are at high risk for developing serious cognitive and socio-emotional disorders and even permanent developmental impairments. These risks are significantly increased if the parents are themselves affected, since adults who have been traumatized by war may be unable to fulfill their parental responsibilities adequately and to create a safe and conducive environment for their children. The family dynamics are often exacerbated by crowded housing environments where there is little or no privacy or personal space to retreat to. Problematic parenting, neglect, and violence against women [23] and children [24, 25] are significantly more frequent in such families. Tragically, children who are already suffering from psychological disorders brought on by societal violence are at particularly high risk of experiencing further maltreatment at home [26], as parents and other caregivers are often overwhelmed by their children’s emotional and behavioral challenges and may respond to these with threats or violence. In many cases, parental abuse arises from helplessness and from a lack of knowledge about positive parenting strategies.

Unsurprisingly, the increased sensitivity seen in PTSD, expressed as heightened irritability, anger, fearfulness, and difficulty in concentration, often manifests itself as increased domestic violence. This association has been found in several studies. Riggs et al. [27] found that significant marital problems such as frequent quarrels, physical violence, or difficulties in intimacy were reported by 70% of Vietnam war veterans who had developed PTSD compared to just 30% of those who had not. Clark et al. [28] found that men who had been directly exposed to political violence had a higher tendency to inflict physical and sexual violence on their wives. Men who have been traumatized by war are more likely to turn to alcohol, which appears to be another crucial risk factor for domestic violence [23–25]. In a survey of couples living in areas of northern Uganda afflicted by civil war [23], 80% of women reported that they had suffered some form of violence at the hands of their partner in the previous year, with 71% reporting physical assaults and 23% reporting sexual assaults. The study also found that the women who had experienced several traumatic events during the war and who showed more severe symptoms of post-traumatic stress were more frequent victims of domestic violence.

**Fig. 1:**
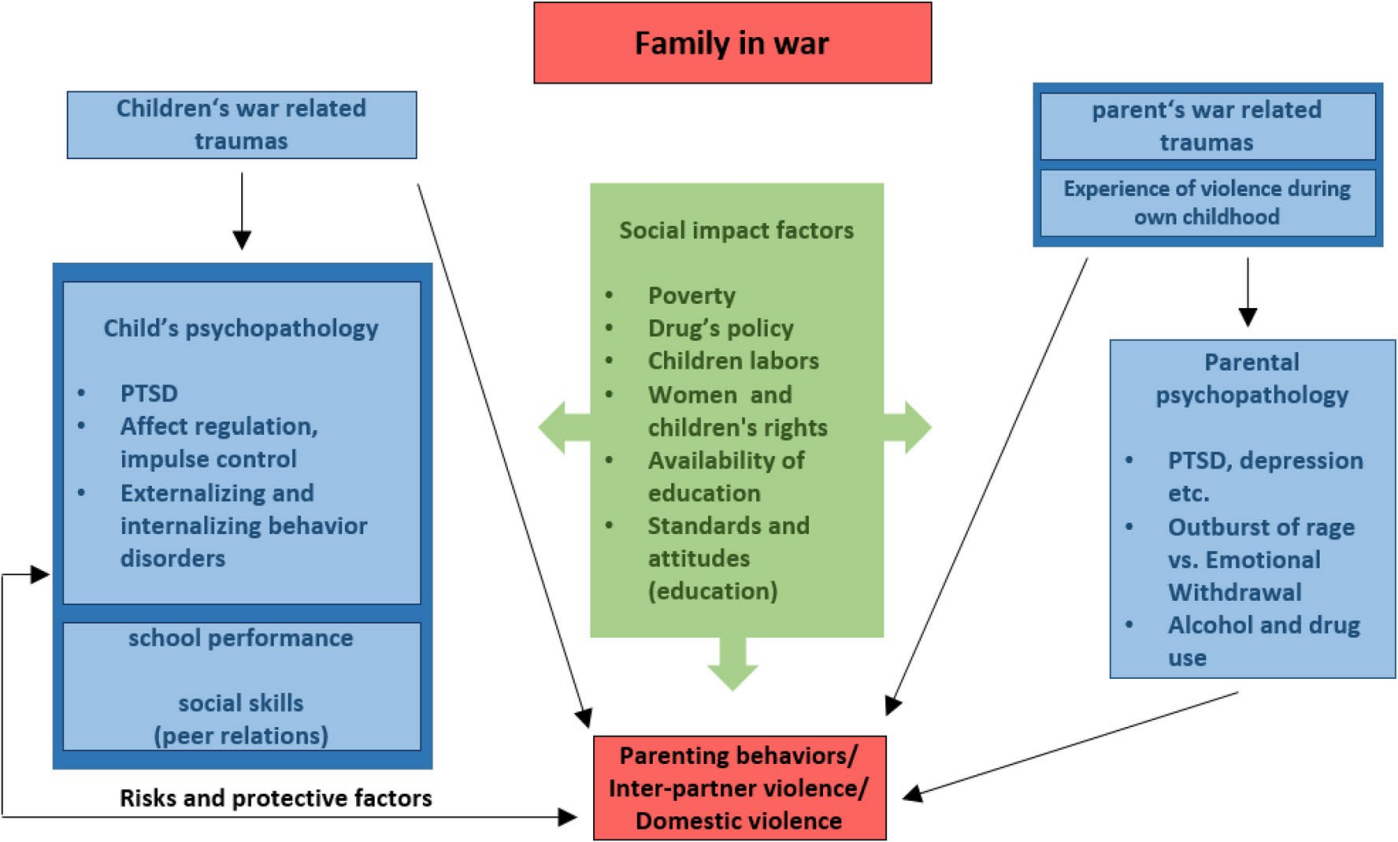
Links between social, individual and familial parameters and domestic violence [31]

In families traumatized by war, domestic violence frequently is directed not just at intimate partners but at children as well. Children and adolescents who have experienced trauma may externalize psychological distress as behavioral problems such as hyperactivity and aggressiveness, and those suffering from PTSD may display various types of incompetence, an inability to concentrate, or refusal to complete schoolwork or household tasks. Parents provoked by these behaviors may attribute them to laziness or defiance, and, whether because of lack of awareness or because of their own stress, may react with threats, verbal abuse, and physical punishment. Conversely, positive parenting practices have been shown to alleviate the problems that children who have been traumatized by war may display, whether these problems are externalized (e.g., aggressive behaviors) or internalized (e.g., anxiety and other emotional burdens). Qouta et al. [29] found such practices to reduce aggressive behaviors in a sample of Palestinian children who had been exposed to military violence, and in a study of families in post-war Sri Lanka, Sriskandarajah et al. [30] found that good parenting provided significant protection against the effects of war trauma on children’s mental health. Figure 1 outlines the relationships between traumatizing experiences, mental stress, and family violence.

## Support for traumatized families

### Strengthening parenting skills

Based on the concerns outlined above, international institutions such as the United Nations High Commissioner for Refugees (UNHCR) and UNICEF have called for the provision of programs to improve the parenting skills of refugees with children, starting as early as during their stay in temporary shelters [32]. The goal is to teach parents strategies that will facilitate interactions with their children and enable them to handle everyday annoyances in a positive way, despite their own traumatization and despite the considerable challenges that their children may be presenting. Up to a certain point in the life of any child, there is no one more important than a parent; and for both children and adolescents, finding ways to deal with stress is best achieved when the home environment is a safe and reliable place where limits and clear rules are defined and shared by all members. Thus, the provision of programs that can provide education on the basic principles and rules of “positive” parenting is vitally important.

A large barrier to be surmounted in these programs is cultural dissimilarities, as the majority of refugees settling in Europe are coming from countries in which values and approaches to family life are very different. Typically, extended family is of high importance and social networks are very family-centered. However, most migrants arrive without their entire families and their contact with close relatives is limited to communicating over the internet, which diminishes the support they can receive from them. Further, the family-centered values in the country of origin often include authoritarian parenting styles in which physical punishment is prevalent. These standards are likely to clash with the prevailing ones held by the host country; certainly, this is often the case in Germany, where an authoritative rather than an authoritarian parenting style is preferred, and corporal punishment has been legally outlawed since 2000 (see the expert report on this topic by the Scientific Advisory Board: Wissenschaftlicher Beirat für Familienfragen [33]). Accordingly, many refugees go through a “culture shock curve” [34], whereby the hope and optimism that prevailed upon their arrival in the host country give way to disillusionment and a negative view of the new and alien environment, and this can drive them to return to the familiar values and traditions of their own culture. Upheavals within the family system can worsen tensions; for example, parents may be distressed by being unable to adequately fulfill the role of provider, or by needing their children to sometimes take on the parent role in matters such as handling interactions with authorities since their children have learned the language more quickly. During this phase in particular, support is urgently needed [34, 43].

### Treatment of trauma-related disorders

Refugee families in need, especially those with one or more members suffering from a trauma-related disorder, would benefit from immediate access to health care services and other targeted support services that can provide relief to the family system as a whole. Normally, when a child receives treatment, the inclusion of the parents is necessary; similarly, when the service recipient is an adult (for example, after a suicide attempt), it is highly recommended that the entire family receive psychological support and be involved in the treatment. Numerous studies have shown that certain types of psychosocial treatments, including cognitive behavioral therapy, eye movement desensitization and reprocessing (EMDR), and narrative exposure therapy, can be highly effective in helping people who have experienced traumatic events or who are in crisis situations. Group interventions, which can be provided in classrooms or daycare facilities, have also been shown to have positive effects [4, 35].

Unfortunately, the German health system is insufficiently prepared for the treatment of so many traumatized refugees with mental disorders. By law, the right of asylum seekers to government-covered health care is limited during the first 15 months after arrival and is restricted to treatment of acute illness or pain. Psychological disorders are usually not considered to meet these criteria. The health care system cannot even meet the demand for psychotherapy services for native-born Germans, with wait-lists for treatment in almost every area. With regard to facilities that offer specialized treatment for refugees with PTSD, there are 23 such centers with a capacity to treat approximately 10,000 patients per year [15], but an estimated 250,000 placements are needed. Thus, while psychiatric and psychotherapeutic services for refugee families do exist, access to them on a broad scale is lacking.

Language barriers present a special challenge in providing psychotherapy to refugees, and sessions can usually only be conducted with the help of interpreters. However, funding of these services is generally not guaranteed, and measures are lacking for the proper training and supervision of translators and interpreters in order to ensure good quality of their work, without which the therapy cannot succeed. In addition, the therapists who are providing the treatment usually have little understanding of the cultural background of their refugee patients, and the patients themselves may hold (culturally-based) feelings of guilt and shame around being diagnosed with a psychological disorder [15, 36].

### Creating a supportive environment

Apart from the provision of formal psychosocial interventions, there are other steps that can be taken to support the successful integration of refugee children and adolescents into the society of the host country. In particular, attendance at schools and daycare centers is an important prerequisite. In a study of more than 4500 adult refugees, many of them from Syria, Afghanistan, and Iraq, Gambaro et al. [37] found that among the children in this sample, over 94% who were of primary school age had attended school in the previous year (although unfortunately only half of these had received extra support for learning the language), while those aged 3 to 6 years had attended daycare facilities at almost the same rate as the German average. However, it is not possible to reach qualitative conclusions about integration based on attendance numbers alone. In the youngest age group (0 to 3 years), the pattern was more unequal, with 15% attending daycare compared to 28% of all children of the same age group in Germany, even though this is the age group that would see the most benefit from language exposure and integration.

With respect to professionals who are involved in the care of refugees or who are working in educational institutions where refugee children are likely to be enrolled, measures are needed to broaden their knowledge of the special difficulties facing these children’s families and to improve their ability to interact with them, so that they can provide the best possible support [38]. The findings of two recent surveys done in Germany underscore the urgent need for such measures, with the majority of both teachers [39] and daycare workers [40] reporting that they do not feel properly prepared to handle the needs of refugee children. Few of the training programs for teachers in Germany address the challenges of an immigration society, such as the provision of extra language instruction or dealing with issues of cultural diversity, and there are insufficient numbers of supporting professionals such as school psychologists or psychotherapists.

## Conclusion

The recommendations developed by the Advisory Council regarding how best to address the needs of traumatized refugee families are summarized below.

1. *Early recognition of psychosocial risks* Individuals from war zones are at markedly increased risk of developing post-traumatic disorders, which may result in dysfunctional behaviors that complicate the ability to cope with social and family life. Provision of early counseling, aid, and support is vital, including access to education and to stimulating forms of leisure activities, and teaching of strategies to relieve stress in everyday life.
2. *Provision of family*–*friendly living accommodations* The temporary housing provided for refugee families should ensure access to privacy, and should have measures in place to protect against menaces such as sexual harassment and other forms of sexual violence. High levels of noise and other types of stimulation should be controlled to support proper sleeping conditions, as “sleeping hygiene” is recognized as being important for the recovery of mental stability. Being settled in living accommodations that are seen as intact and secure can contribute substantially to well-being and psychological stabilization.
3. *Provision of counseling services to strengthen parenting competencies* Parents who have been traumatized may need help to regain or strengthen their parenting competencies, but this assistance must be culturally sensitive since being confronted with foreign views regarding education, parenting, and family life in general often leads to culture shock. The adverse responses to this shock may involve depression, resignation, child neglect, or symptoms of PTSD, which in turn may lead to domestic violence including against children. Assistance should take the form of encouraging non-physical forms of discipline and the introduction of alternative parenting approaches that involve raising children lovingly, consistently, and non-violently. The principle of authoritative parenting, which provides children with “freedom within limits”, has proved to be beneficial in many ways, helping children to reach their development potential, strengthening family relationships, and alleviating adverse consequences in the event of extraordinary stress or trauma (see the report of the Scientific Advisory Board on this topic: Wissenschaftlicher Beirat für Familienfragen [33]). Witt et al. [41] found that the banning of corporal punishment in schools in Germany in 2000 led to a lasting change in social attitudes in the German population, indicating that a change in such values is possible.
4. *Access to a wide range of support services* To improve the mental health and well-being of all family members, diverse support services are needed that draw on existing, cost-effective programs whose effectiveness is backed by scientific knowledge. These programs must meet the following requirements:

- Evidence-based.
- Culturally sensitive.
- Wide and flexible availability.
- Additional support for individuals who are helping refugees.
  Access to professional counseling services, such as the teaching of positive parenting strategies, is limited due to language problems and a serious shortage of trained interpreters. One promising solution is to use low-cost technology approaches such as online programs that are provided also in the language spoken by the recipients. The use of IT technology is cost-efficient, independent of location and time, and can be easily installed on devices such as Smartphones. Programs that are offered online can be adapted to individual needs, anonymity is guaranteed, and stigmatization, which is particularly likely to occur in mixed-sex groups, is avoided. Forms of online interventions have been proven to be very effective in addressing various psychological problems and disorders, with some found to be as effective as face-to-face interventions [42].

5. *Training of people who work with or treat traumatized refugees* The audience for this type of education would include preschool workers, teachers, and professionals and volunteers who work with refugee families. Given the demanding schedules of many of these personnel, information should be provided in short sessions and/or in the form of e-learning modules of short duration. The training should include education about emotional and behavioral disorders that are often seen in adults and children who have fled war zones, including depressive disorders, anxiety disorders, and various trauma-related disorders, and provide practical advice on how to help troubled children in everyday life. Similarities and differences in views on religion, family life, and education should be discussed. To accomplish this, teaching materials should be developed that can be easily adapted to specific circumstances and translated into different languages (German, English, Arabic, Turkish, etc.) for use by trained personnel.
6. *Expanded training and supervision of translators and interpreters* As language barriers are among the biggest hurdles to accessing health care services and other supports, including psychotherapeutic services, translators and interpreters are critical for facilitating access to psycho-educational programs. Sufficient funding for language services must be secured for all members of refugee families, and the providers of these services must be trained so that they can also act as cultural mediators.
7. *Full and immediate access to education for children and adolescents* Children caught up in war and flight may have been deprived of an education for years, and need rapid access to schooling facilities to compensate for what they have missed. Participation in preschool and primary school enables them to integrate more easily into the mainstream society, and extra instruction in the new language is essential [37]. Easy and immediate access to language and education programs should be made available to parents as well.
8. *Full and immediate access to health care for children and adolescents* This access should be granted immediately upon entry to the host country, regardless of current legal residency status. In Germany, the current focus in the health care system on crisis management rather than prevention, as well as the heterogeneity of legal regulations around access to care, have resulted in numerous problems and uncertainties for both providers and beneficiaries. Steps should be taken to allow all refugee children and adolescents, if not their parents, the same comprehensive access to care that is available to their German peers, including referral to further services when necessary.
9. *Provision of screening tools to identify intervention requirements* To plan appropriate interventions, health professionals should have easily applicable screening instruments that enable them to detect both possible psychological problems (in particular those such as suicidality or addictions) and resources in refugee patients [43, 44]. These instruments should be available in different languages. Also, because of gender differences in the risk of becoming a victim of violence, as well as the fact that gender differences in the emotional and behavioral consequences of victimization should be taken into account when conducting a diagnosis, screening questionnaires should allow for gender-specific standard values.
